# Quality of Life in Adults with Eating Disorders

**DOI:** 10.3390/bs16071174

**Published:** 2026-07-11

**Authors:** Renee D. Rienecke, Timothy D. Brewerton, Alan Duffy, Thomas Joiner, Jamie Manwaring, Philip S. Mehler, Dan V. Blalock

**Affiliations:** 1 Eating Recovery Center|Pathlight Mood & Anxiety Centers, Denver, CO 80230, USA; 2 Department of Psychiatry and Behavioral Sciences, Northwestern University, Chicago, IL 60611, USA; 3 Department of Psychiatry and Behavioral Sciences, Medical University of South Carolina, Charleston, SC 29425, USA; 4 Department of Psychology, Florida State University, Tallahassee, FL 32304, USA; 5 School of Medicine, University of Colorado, Denver, CO 80045, USA; 6 Center of Innovation to Accelerate Discovery and Practice Transformation, Durham Veterans Affairs Medical Center, Durham, NC 27705, USA; 7 Department of Psychiatry and Behavioral Sciences, Duke University School of Medicine, Durham, NC 27701, USA

**Keywords:** quality of life, eating disorders, adults, QoL, HRQoL

## Abstract

A dearth of information exists on differences in quality of life across eating disorder diagnoses. The purpose of the current study was to examine quality of life among a large sample of adults with one of six eating disorder diagnoses: anorexia nervosarestricting subtype (AN-R), AN binge/purge subtype (AN-BP), bulimia nervosa (BN), binge eating disorder (BED), avoidant/restrictive food intake disorder (ARFID), and otherwise specified feeding or eating disorder (OSFED). Participants were 1268 adults receiving treatment for an eating disorder across multiple levels of care who completed a measure of quality of life, the WHOQOL-BREF, at admission. Adjusted pairwise comparisons with multiple comparison correction found significant differences among eating disorder groups, even after controlling for age, sex, level of care, body mass index (BMI), and the interaction between BMI and eating disorder diagnosis. Among the five significant findings, four of them involved differences in those with ARFID in comparison to the other eating disorder diagnoses. On the physical health subscale, individuals with BN scored significantly better than did individuals with ARFID. On the psychological subscale, patients with ARFID had significantly better quality of life than those with BN, BED and OSFED. On the social relationships subscale, the BED group scored significantly worse than those with AN-BP. This large study of adults with eating disorders suggests that there are meaningful differences among eating disorder diagnostic groups in quality of life.

## 1. Introduction

Eating disorders are serious psychiatric disorders associated with high rates of medical and psychiatric comorbidities (Udo & Grilo, 2019), high risks of mortality (Chesney et al., 2014), and significantly impaired quality of life (Jenkins et al., 2011). Quality of life is defined in part as a state of well-being across physical, mental, and social aspects of life, and not simply the absence of disease. It is based on an individual’s perception of their position in life in the context of their culture and value systems (WHO, 1998).

Quality of life has been identified as an important treatment target for those with an eating disorder (Hanegraaf et al., 2024), in addition to the goals of weight restoration where necessary and a reduction of eating disorder behaviors, recognizing that true recovery involves more than the absence of the disorder. For example, a commonly-used measure of global quality of life, the World Health Organization Quality of Life-BREF (WHOQOL-BREF) (The WHOQOL Group, 1998), assesses four domains, including physical health, psychological health, social relationships, and one’s environment, reflecting the importance of one’s satisfaction across multiple levels of functioning. General questionnaires such as the WHOQOL-BREF assess quality of life without focusing on what might be driving the level of quality. Health-related quality of life, while a similar construct and often used interchangeably with quality of life (Karimi & Brazier, 2016), generally assesses the specific impact of one’s physical and mental health status on quality of life (Yin et al., 2016). The commonly used Medical Outcomes Trust short form 36-item health survey (SF-36) (Ware & Sherbourne, 1992), for example, focuses on the impact of one’s physical and emotional health on physical, social, and “usual” role activities, bodily pain, general mental health, and vitality. The inclusion of the environmental domain distinguishes the WHOQOL-BREF from traditional health-related quality of life measures such as the SF-36, which focuses more narrowly on health-related functional status.

It has been well established that individuals with eating disorders report poorer health-related quality of life and quality of life than healthy controls (Ágh et al., 2016; Jenkins et al., 2011; Mond et al., 2012). What is somewhat less clear is whether there are differences in health-related quality of life and quality of life among eating disorder diagnoses, as findings are mixed.

### 1.1. Health-Related Quality of Life

Several studies have found no differences between eating disorder diagnostic groups on health-related quality of life (Carta et al., 2014; de la Rie et al., 2005), including a meta-analysis on this topic (Winkler et al., 2014), while others have found worse physical functioning among individuals with binge eating disorder (BED) compared to individuals with other eating disorders, although the BED sample size was fairly small (*n* = 17) (Padierna et al., 2000). Another study found that those with anorexia nervosa (AN) reported the highest levels of health-related quality of life, followed by those with eating disorder not otherwise specified (EDNOS), with those with bulimia nervosa (BN) scoring the poorest (Martín et al., 2017).

### 1.2. Quality of Life

Those with AN-restricting subtype (AN-R) have been found to report higher satisfaction with their social relationships than those with eating disorders characterized by binge eating and/or purging behaviors (Mond et al., 2005).

Among individuals receiving outpatient treatment for an eating disorder, those with EDNOS reported better scores on psychological quality of life than other eating disorder diagnostic groups (Baiano et al., 2014; Bamford & Sly, 2010). A study of adolescents and adults receiving outpatient treatment for an eating disorder found that those with AN-binge/purge subtype (AN-BP) reported worse scores on the work/school quality of life subscale than those with other eating disorder diagnoses (AN-R, BN, and EDNOS), and worse scores on the psychological quality of life subscale than those with AN-R or EDNOS (Ackard et al., 2014).

The use of different questionnaires across studies, the small sample sizes in many studies, and the often interchangeable use of the terms “quality of life” and “health-related quality of life”, combine to make it challenging to arrive at firm, generalizable conclusions regarding differences in quality of life across different eating disorder diagnoses. In addition, there is a lack of research on quality of life among patients with avoidant/restrictive food intake disorder (ARFID). ARFID was added to the category of “feeding and eating disorders” in the *DSM-5* (APA, 2013). Individuals with ARFID may be at a low weight and can present with a number of adverse medical sequelae (Leach et al., 2024; Nitsch et al., 2023), similar to individuals with AN; however, restrictive eating behaviors in ARFID are not driven by the body image concerns or fear of weight gain that are characteristic of other eating disorders. Thus, ARFID and other eating disorders may have very different quality of life characteristics.

A large study examining quality of life among adults with and without symptoms of ARFID found that those with ARFID symptoms reported worse physical and mental quality of life than those without ARFID symptoms (Brownlow et al., 2026). However, this study did not compare those with diagnoses of ARFID to individuals with diagnoses of other eating disorders. The purpose of the current study was to examine quality of life among a large sample of adults with eating disorders across six diagnostic groups, including ARFID, at the onset of treatment to a higher level of care. Given the limitations and mixed findings of previous studies, no *a priori* hypotheses were proffered; rather, this was considered a hypothesis-generating study. Identifying specific areas of quality of life that may be problematic for specific eating disorder groups may point to important targets for treatment and determine avenues for future research to examine possible reasons behind differences in quality of life.

## 2. Method

### 2.1. Participants and Procedure

Participants were 1268 adults receiving treatment at one of 37 networked specialty facilities, all part of a single treatment program offering higher levels of care for an eating disorder between March 2024 and November 2025. Diagnoses were made by Masters’ level clinicians with a semi-structured interview based on the *Diagnostic and Statistical Manual of Mental Disorders—5th edition (DSM-5)* criteria (APA, 2013). Diagnoses were then confirmed by a multidisciplinary team of a board-certified primary care physician, a board-certified psychiatrist, and a therapist using a *DSM-5*-based diagnostic clinical interview. At the IOP level, the therapist is responsible for making diagnoses. Self-report measures were completed at admission. All patients over the age of 18 who completed the quality of life measure at admission, and who provided informed consent at admission for their clinical data to be used for research purposes, were included in the current study; there were no exclusion criteria. As part of the informed consent process, all patients were made aware that their participation or abstention in research would have no impact on their treatment. Demographic data were collected from patient charts. This study was approved by Salus Institutional Review Board.

### 2.2. Measures

The World Health Organization Quality of Life-BREF (WHOQOL-BREF) (The WHOQOL Group, 1998) is a 26-item self-report measure of quality of life. It assesses four domains: physical health (e.g., “Do you have enough energy for everyday life?”), psychological (e.g., “To what extent do you feel your life to be meaningful?”), social relationships (e.g., “How satisfied are you with the support you get from your friends?”), and environment (e.g., “How safe do you feel in your daily life?”), as well as an overall assessment of quality of life and general health (e.g., “How would you rate your quality of life?”). Domains are scored on different scales. The range for the overall assessment is 2–10; the range for physical health is 7–35; the range for psychological is 6–30; the range for social relationships is 3–15; and the range for environment is 8–40. Higher scores indicate a higher quality of life. The WHOQOL-BREF has been shown to have good discriminant validity, content validity, internal consistency, and test–retest reliability (The WHOQOL Group, 1998).

### 2.3. Statistical Analyses

Unadjusted and adjusted linear regressions were conducted for all analyses. Univariate associations predicting the WHOQOL-BREF total and each subscale from patient age, sex, race, body mass index (BMI) on admission, and level of care were examined. Significant predictors of the WHOQOL-BREF total or any subscale were included as covariates in subsequent adjusted models. With regard to BMI, given the differing associations of BMI with eating disorder severity depending on eating disorder diagnosis, the main effect of BMI for any analyses is accompanied by the interaction between BMI and eating disorder diagnosis (Harrell, 2001). The primary predictor was the 6-level categorical variable of eating disorder diagnosis, with pairwise contrasts examined between each pair of eating disorder diagnoses. Given the focus on pairwise comparisons between eating disorder diagnoses, Cohen’s *d* and 95% confidence intervals (95% CIs) are presented as effect sizes. In light of the large number of pairwise comparisons, a Holm multiple-test correction was performed within each outcome (with a functional range of α = 0.0033 to α = 0.05; Aickin & Gensler, 1996). Because each of the quality of life outcomes represented either an overall composite (WHOQOL-BREF overall scale), or distinct subscales with potentially distinct implications, other multivariable analyses such as MANOVA that would create an additional composite across these outcomes were not considered. The results of both the unadjusted and adjusted models are presented both with and without the Holm correction. All analyses were performed in R (version 4.5.2; R Core Team, 2025).

## 3. Results

### 3.1. Descriptive Sample Information

Demographic and clinical characteristics of the sample overall and by eating disorder diagnosis are presented in Table 1. On average, the sample was 27.6 years old (SD = 10.0), 88.2% female sex assigned at birth, 77.3% female-identifying, and 78.7% White race. Among the total sample of 1268 patients, all ED diagnoses were represented, including OSFED (*n* = 594; 46.8%), ANR (*n* = 253; 20.0%), ARFID (*n* = 160; 12.6%), ANBP (*n* = 123; 9.7%), BN (*n* = 102, 8.0%), and BED (*n* = 36, 2.8%).

Age (*p* = 0.02), level of care (*p* < 0.001), and BMI (*p* < 0.001) were all associated with WHOQOL overall quality of life, and sex (*p* < 0.001) was associated with WHOQOL psychological, and thus were used as covariates in subsequent adjusted analyses (including with regard to the interactions between BMI and eating disorder diagnosis). Race was not associated with WHOQOL overall quality of life or any subscales (all *p*’s > 0.10).

### 3.2. Unadjusted ED Diagnosis Differences

Unadjusted associations between each pairwise comparison of eating disorder diagnosis across WHOQOL overall quality of life and each WHOQOL subscale, including effect sizes, 95% CIs, and statistical significance before and after multiple comparison correction, are presented in Table 1 and Supplementary Figure S2. Before multiple comparison correction: WHOQOL overall quality of life was significantly lower in patients with BED than patients with AN-R and patients with BN; WHOQOL environment was significantly lower in patients with ARFID than patients with AN-R and patients with BN, and significantly lower in patients with other specified feeding or eating disorder (OSFED) than patients with AN-R and patients with BN; WHOQOL physical was significantly lower in patients with ARFID than patients with AN-R and patients with BN, significantly lower in patients with OSFED than patients with AN-R and patients with BN, and significantly lower in patients with BED than patients with AN-R and patients with BN; WHOQOL social was significantly lower in patients with BED than patients with AN-BP, patients with AN-R, patients with ARFID, patients with BN, and patients with OSFED; and WHOQOL psychological was significantly lower in patients with AN-BP, patients with AN-R, patients with BED, patients with BN, and patients with OSFED than patients with ARFID, and significantly lower in patients with OSFED than patients with AN-R. Effect sizes for significant pairwise differences between eating disorder diagnoses in unadjusted models without multiple comparison correction ranged from small (*d* = 0.23: WHOQOL physical in patients with OSFED compared to patients with AN-R) to moderate (*d* = 0.73: WHOQOL psychological in patients with ARFID compared to patients with AN-BP).

In unadjusted models after multiple comparison correction: WHOQOL overall quality of life was not significantly different between any pairwise comparison of eating disorder diagnoses; WHOQOL environment remained significantly lower in patients with OSFED than patients with AN-R and patients with BN; WHOQOL physical remained significantly lower in patients with ARFID than patients with AN-R, and remained significantly lower in patients with OSFED than patients with AN-R; WHOQOL social remained significantly lower in patients with BED than patients with AN-R, patients with ARFID, and patients with BN; and WHOQOL psychological remained significantly lower in patients with AN-BP, patients with AN-R, patients with BED, patients with BN, and patients with OSFED than patients with ARFID, and remained significantly lower in patients with OSFED than patients with AN-R. Effect sizes for significant pairwise differences between eating disorder diagnoses in unadjusted models after multiple comparison correction ranged from small (*d* = 0.23: WHOQOL physical in patients with OSFED compared to patients with AN-R) to moderate (*d* = 0.73: WHOQOL psychological in patients with ARFID compared to patients with AN-BP).

### 3.3. Adjusted ED Diagnosis Differences

Adjusted associations between each pairwise comparison of eating disorder diagnosis across WHOQOL overall quality of life and each WHOQOL subscale, including effect sizes, 95% CIs, and statistical significance before and after multiple comparison correction, are presented in Table 2 and Supplementary Figure S1. Before multiple comparison correction: WHOQOL overall quality of life was significantly lower in patients with BED than patients with BN and patients with OSFED; WHOQOL environment was significantly lower in patients with ARFID, patients with BN, and patients with OSFED than patients with BN; WHOQOL physical was significantly lower in patients with ARFID than patients with BN and patients with OSFED, and significantly lower in patients with OSFED than patients with BN; WHOQOL social was significantly lower in patients with ARFID, patients with BED, patients with BN, and patients with OSFED than patients with AN-BP, significantly lower in patients with BED, patients with BN, and patients with OSFED than patients with AN-R, and significantly lower in patients with BED than patients with ARFID; and WHOQOL psychological was significantly lower in patients with AN-R, patients with BED, patients with BN, and patients with OSFED than patients with ARFID. Effect sizes for significant pairwise differences between eating disorder diagnoses in adjusted models without multiple comparison correction ranged from small (*d* = 0.20: WHOQOL physical in patients with OSFED compared to patients with ARFID) to very large (*d* = 1.98: WHOQOL social in patients with BED compared to patients with AN-BP).

In the adjusted models after multiple comparison correction, WHOQOL overall quality of life was not significantly different between any pairwise comparison of eating disorder diagnoses; WHOQOL environment was not significantly different between any pairwise comparison of eating disorder diagnoses; WHOQOL physical remained significantly lower in patients with ARFID than patients with BN; WHOQOL social was significantly lower in patients with BED than patients with AN-BP; and WHOQOL psychological was significantly lower in patients with BED, patients with BN, and patients with OSFED than patients with ARFID. Effect sizes for significant pairwise differences between eating disorder diagnoses in adjusted models after multiple comparison correction ranged from moderate (*d* = 0.51: WHOQOL physical in patients with BN compared to patients with ARFID) to very large (*d* = 1.98: WHOQOL social in patients with BED compared to patients with AN-BP). The WHOQOL scores, rank ordered for each subscale by eating disorder diagnosis, are presented in Table 3 and Table 4.

## 4. Discussion

The purpose of the current study was to examine quality of life among a large sample of adults with eating disorders. Given the limitations and mixed findings of previous studies, there were no *a priori* hypotheses. Adjusted pairwise comparisons with multiple comparison correction found several significant differences among eating disorder groups, even after controlling for age, sex, level of care, BMI, and the interaction between BMI and eating disorder diagnosis. It is notable that among the five significant findings, four of them involved differences in those with ARFID in comparison to the other eating disorder diagnoses. The adjusted results will be reviewed first, followed by results from unadjusted comparisons, which may shed additional light on patterns of findings but should be considered exploratory.

### 4.1. Adjusted Results

*Overall Quality of Life*. No differences were found between eating disorder groups on overall quality of life. As the overall quality of life score seemed to obscure some of the findings that emerged on the different subscales, it is possible that it may not be an informative measure of quality of life on its own. Notably, the overall quality of life score is based on only two items; its utility over and above the WHOQOL-BREF subscales should be determined in future studies.

*Physical Health Quality of Life*. Individuals with BN scored significantly better on the physical health subscale than did individuals with ARFID. Although all types of eating disorders have been found to have impaired quality of life, these findings are somewhat unexpected, as those with binge-purge diagnoses and comorbidities have generally been found to have worse quality of life compared to other eating disorder diagnoses (Ackard et al., 2011; DeJong et al., 2013; Gonzalez-Pinto et al., 2004). However, these studies did not utilize the WHOQOL-BREF and also did not include patients with ARFID. Patients with ARFID can be significantly underweight and can present with a number of medical complications, as it is often physical discomfort or pain that leads to restriction (Cooney et al., 2018; Leach et al., 2024; Nitsch et al., 2023).

Although patients with ARFID had a BMI in the “normal” category in this study, medical complications may have accounted for worse physical health quality of life, as significant medical complications can also occur among individuals with ARFID who are in the “normal” or “overweight” weight range (James et al., 2024). Information on medical complications for patients was not available in the current study and would be an additional important area for future research. It is worth noting that those in the ARFID group and those in the BN group were similar ages in this study. However, ARFID has a much earlier age of onset than BN, with some reports of ARFID beginning at infancy, compared to the average age of onset of 18 for BN (Bertrand et al., 2024; Cañas et al., 2021; Krom et al., 2019; Volpe et al., 2016). More years suffering from an eating disorder could result in additional physical complications and worse physical health. The current study did not have information on duration of illness; this would be a fruitful area for future research.

Treatment for ARFID requires nutritional rehabilitation and, for some, weight restoration (Fonseca et al., 2024). Future research should assess physical health quality of life before and after treatment to determine whether this aspect of quality of life improves with nutritional intervention.

*Psychological Quality of Life*. Patients with ARFID had significantly better psychological quality of life than several other eating disorder groups. This is in contrast to findings from a large Australian study that found that mental health-related quality of life was particularly poor among those with ARFID (Hay et al., 2017). However, it is not clear to what extent mental health-related quality of life is related to psychological quality of life as measured by the WHOQOL-BREF, although some studies have shown positive but weak correlations between the WHOQOL-BREF and other health-related quality of life measures (Hsiung et al., 2005; Huang et al., 2006). To the authors’ knowledge, there are no published peer-reviewed studies that have specifically used the WHOQOL-BREF in patients with ARFID.

Two large population-based studies examined quality of life in adults with ARFID symptoms and although they reported reduced mental and physical quality of life, they did not identify the specific quality of life measure utilized (Brownlow et al., 2026; Flack et al., 2026). In addition, none of these studies compared patients with ARFID to those with other eating disorder diagnoses. ARFID is not characterized by the overvaluation of weight and shape that is observed, or is even a required diagnostic criterion, in other eating disorder diagnoses (APA, 2013). It is possible that the absence of that symptom results in less psychological distress for those with ARFID. However, patients with ARFID did not differ from patients with AN in this regard; patients with AN have significant body shape and weight concerns (APA, 2013), but the disorder is also often ego-syntonic (Gregertsen et al., 2017), which may have impacted quality of life reporting for patients with AN.

Although better psychological quality of life was found among those with ARFID compared to other eating disorder diagnoses, these findings should be interpreted cautiously, as information on psychiatric comorbidities, such as autism spectrum disorder, which is often comorbid with ARFID (Sader et al., 2025), was not available for the current study. Further, presentations of ARFID are heterogeneous; it is possible that better psychological quality of life is found among some presentations but not others. Future qualitative studies may shed further light on these findings; if psychological quality of life is indeed better among those with ARFID, is it due to the lack of body/shape/weight concerns? A qualitative meta-synthesis of fifteen studies including individuals with various eating disorders (primarily AN), investigated the eating disorder “voice” (Tragantzopoulou et al., 2024). Patients described this voice as becoming more controlling and hostile as the disorder progressed, and compared dealing with this voice as being in an internal battle, associated with significant negative emotions: “The voice of anorexia constantly tells me, ‘You can’t do that, you shouldn’t do this, you’re not good enough, you’re not thin enough, no one will ever want you, you have no friends, you’re boring, you’re ‘damaged goods’, you have to exercise or you’re a failure” (p. 7). One might imagine the toll that this internal battle could take on someone over time, likely resulting in reduced psychological quality of life.

Whether those with ARFID experience a similar voice is not a topic that has received attention in the eating disorder literature. The absence of such a voice could be protective of psychological health. It is worth noting here that those with AN did not differ significantly on psychological quality of life from those with ARFID. Again, this could be due to the ego-syntonic nature of AN; the meta-synthesis found that patients found the eating disorder “voice”, in addition to being intrusive and controlling, to be comforting and protective at the same time. Psychological quality of life could be an important treatment target for those with eating disorders; better understanding the reasons behind better psychological quality of life in certain eating disorder groups is an important area for future research.

*Social Relationships Quality of Life*. On the social relationships subscale, those with BED scored worse than those with AN-BP, with the largest effect size of all results (*d* = 1.98). It is possible that this is due to the significantly higher mean BMI in the BED group. Obesity has been linked to impairments in social relationships (Carr & Friedman, 2006), poorer quality of social life compared to non-obese control groups (Albano et al., 2019), and is associated with elevated levels of loneliness (Timkova et al., 2025) and depression (Luppino et al., 2010), which could have a negative impact on relationships. Although speculative, this could also be explained by experiences of weight bias and stigma from others (Puhl & Heuer, 2009; Rieger et al., 2010), which have been shown to be associated with worse mental- and physical-health-related quality of life (Pearl & Puhl, 2018). Social support is an important aspect of good mental health (Harandi et al., 2017); ensuring the presence or development of a positive support system should be a part of mental health treatments, with sensitivity for those who may experience stigma and internalized weight bias. Effective interventions targeting weight bias internalization exist, and are most effective when related factors, such as body image, self-esteem, and feelings of shame are also addressed (Ramsamy et al., 2024). These interventions should be considered as part of a comprehensive treatment approach for those individuals in larger bodies who are struggling with poor social relationships or lack of social support.

*Environment Quality of Life*. No significant differences were found between eating disorder diagnoses on the environment subscale of the WHOQOL-BREF.

### 4.2. Unadjusted Results

Because the pattern of results differed meaningfully in unadjusted associations, and there is clinical utility in knowing observed differences in quality of life by diagnosis alone, irrespective of other factors that are difficult to consider simultaneously in clinical presentations, patterns in unadjusted associations worth further investigation are also discussed.

*Overall Quality of Life*. Those with BED scored lower than several other eating disorder groups on overall quality of life. Again, it is possible that the higher BMI among the individuals with BED explains these findings, as a higher BMI has been found to be associated with greater impairment in certain aspects of quality of life (Rieger et al., 2005). It is important, however, to not attribute differences in quality of life solely to BMI, as patients with obesity and BED have been shown to have poorer health-related quality of life than patients with obesity without BED (Baiano et al., 2014; Perez & Warren, 2012; Vancampfort et al., 2014), suggesting that the presence of this eating disorder is independently negatively associated with health-related quality of life.

A study examining the loss of control eating associated with binge-spectrum disorders found that those individuals (bariatric surgery candidates, members of a non-surgical weight loss support group, and community respondents) who were most disturbed by their loss of control eating reported higher symptoms of depression and poorer mental health-related quality of life; further, this association was highest among those meeting the full diagnostic criteria for BED (Colles et al., 2008), suggesting that loss of control eating over and above BMI may negatively impact health-related quality of life. It is worth emphasizing here that the terms “quality of life” and “health-related quality of life” are not being used interchangeably; “health-related quality of life” is referred to when that is the term and associated measure used in the specific study.

Further evidence for the negative impact of loss of control on health-related quality of life can be found when comparing objective binge episodes, subjective binge episodes, and objective overeating. Objective binge episodes, in which a large amount of food is consumed accompanied by a sense of loss of control, are characteristic of binge/purge spectrum disorders. Subjective binge episodes occur when a sense of loss of control over eating occurs in the absence of the consumption of an objectively large amount of food. Objective overeating occurs when a large amount of food is consumed without an accompanying sense of loss of control. Both objective and subjective binge episodes have been found to be associated with impaired mental- and physical-health-related quality of life, while objective overeating was not, suggesting that it is not the amount of food eaten that causes distress, but the experience of being out of control (Vallance et al., 2011). Thus, repeated experiences of loss of control eating likely contribute to poor health-related quality of life over and above BMI. Future research may seek to further investigate quality of life among those with BED, as the small sample size in the current study encourages cautious interpretation of any conclusions based on this group.

*Physical Health Quality of Life*. Those with AN-R and BN both reported significantly better physical health than those with ARFID, BED, and OSFED. As above, it is possible that BMI may impact the physical health of those with BED (Casaes et al., 2024; Rieger et al., 2005). Although the BMIs of those with ARFID and OSFED fell into the “normal” and “overweight” categories, respectively, those with ARFID, even without being underweight, can suffer from nutritional deficiencies (APA, 2013) or medical concerns (James et al., 2024) that could adversely impact their physical health. The higher estimation of quality of life for those with AN-R, even though they were at low BMIs, could be due to the ego-syntonic nature of AN (Gregertsen et al., 2017) or the lack of insight often associated with this disorder (Konstantakopoulos et al., 2011). Still, without assessing ego-syntonicity, this remains speculative. The heterogeneous category of OSFED makes it challenging to generate a hypothesis about why they had worse physical health quality of life than those with AN-R or BN.

*Psychological Quality of Life*. Patients with ARFID scored significantly better on psychological quality of life than every other diagnostic group. When considering the covariates that were accounted for in adjusted analyses, it is interesting that this patient group had the lowest percentage of cisgender females and the highest percentage of non-binary patients, raising the question of whether patients’ sex had an impact on the reporting of psychological quality of life.

*Social Relationships Quality of Life*. The BED group scored worse on social relationship quality of life than every other diagnosis. This could be why interpersonal psychotherapy (IPT) has been found to be helpful for BED, not just in terms of reducing binge eating, but also in improving interpersonal relationships, particularly when IPT is delivered in a group format (Aires et al., 2025).

*Environment Quality of Life*. Those with ARFID and those with OSFED rated their environmental quality of life as significantly worse than those with AN-R and BN. One might have expected that those with a higher degree of purging behaviors would have significantly worse environmental stressors (DeJong et al., 2013; Hay et al., 2017), but that was not found here.

### 4.3. Strengths and Limitations

There are several notable limitations to this research, which focused specifically on differences between eating disorder diagnoses. There was not a non-eating disorder comparison group. It was also not possible to assess the influence of comorbid psychiatric disorders on quality of life, which have been known to adversely influence quality of life in patients with eating disorders. For example, individuals with post-traumatic stress disorder (PTSD), autism spectrum disorder, and/or major depressive disorder have been found to have low scores on WHOQOL-BREF domains (Araujo et al., 2014; Johansen et al., 2007; Lefebvre et al., 2021). Importantly, patients with eating disorders and PTSD have been found to have significantly lower measures of quality of life and health-related quality of life compared to those with eating disorders without PTSD (Brewerton et al., 2020, 2025). Future studies that examine the influence of trauma history and comorbid psychiatric disorders on quality of life across eating disorders are warranted.

Another limitation of this study was the relatively smaller sample of patients with BED and the uneven sample sizes across diagnoses. Interpretation of the results was also complicated by the different impacts of the various covariates, differences in the reliability of the effect sizes (as seen by varying sizes of 95% CIs for these effect sizes), and differential effects of multiple comparison corrections. Thus, any differences found in the current study are worth further investigation, but the adjusted results with multiple comparison correction are most likely to be actual differences. Additionally, because the effect sizes for adjusted analyses were necessarily calculated using marginal means and residual standard errors, they are not directly comparable to similar unadjusted effect sizes. Moreover, the BMI-by-eating disorder diagnosis interaction was significant in many cases, representing heterogeneity of effects across levels of BMI. Future studies should perform more targeted examinations within individual diagnoses, and examine how variation in BMI impacts quality of life within these more diagnostically homogeneous groups. In addition, participants in the current study were adults seeking treatment at a higher level of care and findings may not be generalizable to children and adolescents, or outpatient or community samples. For this study, it was not possible to separate those with OSFED into the more specific *DSM-5* subcategories. This resulted in a heterogeneous diagnostic group that could have obscured clinically meaningful differences. Examining the OSFED group according to its subcategories will be an important area for future research. In addition, quality of life can be impacted by a number of factors that this study was unable to assess. In addition to the aforementioned psychiatric and medical comorbidities, many other variables, such as duration of illness, family relationships, social support, history of relapse, etc., can all impact quality of life. A more comprehensive overview of all these factors, and the possible reasons behind differences in eating disorder diagnoses found in the current study, is beyond the scope of this paper, but remains an important area for future research. Strengths of this study include the large sample size, the inclusion of a number of eating disorder diagnoses including ARFID, the ability to separate patients with AN into both subtypes, and a validated measure of general quality of life.

## 5. Conclusions

Several significant differences in WHOQOL-BREF domains across eating disorder diagnoses were found in the current study. Most of these differences involved ARFID, in that patients with ARFID had significantly better psychological quality of life than several other diagnostic groups, suggesting the possibility that the absence of significant body, shape, or weight concerns may contribute to better psychological quality of life among those with ARFID. However, those with ARFID reported significantly worse physical health quality of life than those with BN, which may be due to the medical complications often found among individuals with ARFID. The final significant difference was that patients with BED reported significantly worse social relationship quality of life than those with AN-BP. Findings from the current study suggest that there are meaningful differences between certain eating disorder diagnoses on quality of life, which may provide important targets for treatment.

## Figures and Tables

**Table 1:** Demographic and clinical characteristics by eating disorder diagnosis.

Variable at Admission	AN-R (*n* = 253)	AN-BP (*n* = 123)	BN (*n* = 102)	BED (*n* = 36)	ARFID (*n* = 160)	OSFED (*n* = 594)	Total (*n* = 1268)
**Age (M, SD)**	26.3 (10.7)	28.2 (10.4)	27.5 (9.32)	34.0 (13.7)	27.1 (9.73)	27.7 (9.37)	27.6 (10.0)
**BMI (M, SD)**	16.3 (1.41)	16.7 (1.38)	29.2 (10.9)	44.2 (14.4)	21.2 (6.33)	28.1 (8.81)	24.3 (9.84)
**Gender (*n*, %)**							
Female	219 (86.6%)	103 (83.7%)	84 (82.4%)	23 (63.9%)	98 (61.3%)	453 (76.3%)	980 (77.3%)
Male	11 (4.3%)	7 (5.7%)	8 (7.8%)	9 (25.0%)	32 (20.0%)	33 (5.6%)	100 (7.9%)
TGF	3 (1.2%)	1 (0.8%)	2 (2.0%)	3 (8.3%)	2 (1.3%)	10 (1.7%)	21 (1.7%)
TGM	3 (1.2%)	5 (4.1%)	2 (2.0%)	1 (2.8%)	5 (3.1%)	24 (4.0%)	40 (3.2%)
Non-binary	11 (4.3%)	2 (1.6%)	5 (4.9%)	0 (0.0%)	17 (10.6%)	53 (8.9%)	88 (6.9%)
Other	0 (0.0%)	2 (0.8%)	1 (1.0%)	0 (0.0%)	4 (2.5%)	11 (1.9%)	18 (1.4%)
Prefer Not to Disclose	4 (1.6%)	5 (4.1%)	0 (0.0%)	0 (0.0%)	2 (1.3%)	10 (1.7%)	21 (1.7%)
**Race (*n*, %)**							
White	213 (84.2%)	108 (87.8%)	74 (72.5%)	28 (77.8%)	131 (81.9%)	444 (74.7%)	998 (78.7%)
Hispanic/Latinx	13 (5.1%)	3 (2.4%)	9 (8.8%)	2 (5.6%)	13 (8.1%)	44 (7.4%)	84 (6.6%)
Black/African American	2 (0.8%)	2 (1.6%)	7 (6.9%)	1 (2.8%)	3 (1.9%)	48 (8.1%)	63 (5.0%)
Asian	9 (3.6%)	4 (3.3%)	2 (2.0%)	3 (8.3%)	2 (1.3%)	14 (2.4%)	34 (2.7%)
American Indian/Alaska Native	0 (0.0%)	1 (0.8%)	0 (0.0%)	0 (0.0%)	0 (0.0%)	3 (0.5%)	4 (0.3%)
Native Hawaiian/Other Pacific Islander	0 (0.0%)	1 (0.8%)	0 (0.0%)	0 (0.0%)	1 (0.6%)	1 (0.2%)	3 (0.2%)
Mixed Ethnicity	5 (2.0%)	0 (0.0%)	3 (2.9%)	0 (0.0%)	3 (1.9%)	3 (0.5%)	14 (1.1%)
Other/Prefer Not to Disclose	11 (4.3%)	4 (3.3%)	7 (6.9%)	2 (5.6%)	7 (4.4%)	37 (6.2%)	68 (5.4%)
**Level of Care (*n*, %)**							
IP	114 (45.1%)	69 (56.1%)	12 (11.8%)	0 (0.0%)	30 (18.8%)	70 (11.8%)	295 (23.3%)
RES	86 (34.0%)	36 (29.3%)	30 (29.4%)	8 (22.2%)	36 (22.5%)	210 (35.4%)	406 (32.0%)
PHP	39 (15.4%)	13 (10.6%)	36 (35.3%)	20 (55.6%)	68 (42.5%)	196 (33.0%)	372 (29.3%)
IOP	14 (5.5%)	5 (4.1%)	24 (23.5%)	8 (22.2%)	26 (16.3%)	118 (19.9%)	195 (15.4%)
**WHOQOL-BREF (M, SD)**							
Overall QoL (2–10)	5.40 (1.71)	5.29 (1.79)	5.62 (1.71)	4.75 (1.68)	5.28 (1.71)	5.35 (1.82)	5.35 (1.77)
Physical Health (7–35)	22.7 (5.39)	21.8 (5.64)	22.8 (5.46)	20.4 (5.79)	21.1 (5.31)	21.4 (5.34)	21.7 (5.43)
Psychological (6–30)	15.1 (4.46)	13.8 (5.14)	14.5 (3.78)	14.5 (4.51)	17.0 (3.80)	13.9 (4.18)	14.6 (4.39)
Social Relationships (3–15)	9.74 (2.57)	9.43 (2.73)	9.76 (2.49)	8.22 (2.58)	9.68 (2.45)	9.36 (2.63)	9.48 (2.60)
Environment (8–40)	30.8 (5.86)	29.5 (5.86)	30.6 (5.50)	28.8 (7.14)	29.1 (5.30)	28.7 (6.10)	29.4 (5.97)

Note. AN-R = anorexia nervosa restricting subtype; AN-BP = anorexia nervosa binge/purge subtype; BN = bulimia nervosa; BED = binge eating disorder; ARFID = avoidant/restrictive food intake disorder; OSFED = other specified feeding or eating disorder; BMI = body mass index; TGF = transgender female; TGM = transgender male; IP = inpatient; RES = residential; PHP = partial hospitalization program; IOP = intensive outpatient program; QoL = quality of life.

**Table 2:** Adjusted pairwise comparisons of quality of life between eating disorder diagnoses.

WHOQOL Scale	Cohen’s *d*	*d* 95% CI	*b*-Value	*t*-Value	*p*-Value
**Overall**					
ANBP vs. ANR	−0.46	[−1.69, 0.76]	−0.44	−0.74	0.46
ANBP vs. ARFID	−0.12	[−1.14, 0.90]	−0.11	−0.23	0.82
ANBP vs. BED	0.42	[−0.72, 1.57]	0.40	0.72	0.47
ANBP vs. BN	−0.38	[−1.41, 0.64]	−0.36	−0.73	0.46
ANBP vs. OSFED	−0.25	[−1.26, 0.76]	−0.23	−0.48	0.63
ANR vs. ARFID	0.34	[−0.39, 1.08]	0.32	0.91	0.36
ANR vs. BED	0.89	[−0.02, 1.80]	0.85	1.91	0.06
ANR vs. BN	0.07	[−0.66, 0.82]	0.07	0.21	0.83
ANR vs. OSFED	0.21	[−0.50, 0.93]	0.20	0.58	0.56
ARFID vs. BED	0.54	[−0.04, 1.13]	0.52	1.81	0.07
ARFID vs. BN	−0.26	[−0.53, 0.01]	−0.25	−1.88	0.06
ARFID vs. OSFED	−0.12	[−0.32, 0.06]	−0.12	−1.29	0.20
BED vs. BN	−0.81	[−1.41, −0.20]	−0.77	−2.62	0.09 ^†^
BED vs. OSFED	−0.67	[−1.25, −0.10]	−0.64	−2.30	0.02 ^†^
BN vs. OSFED	0.13	[−0.09, 0.36]	0.12	1.14	0.25
**Physical Health**					
ANBP vs. ANR	0.22	[−1.00, 1.45]	0.21	0.35	0.72
ANBP vs. ARFID	0.75	[−0.26, 1.77]	0.71	1.45	0.15
ANBP vs. BED	0.75	[−0.39, 1.90]	0.71	1.28	0.20
ANBP vs. BN	0.24	[−0.78, 1.27]	0.22	0.45	0.65
ANBP vs. OSFED	0.55	[−0.45, 1.56]	0.52	1.07	0.28
ANR vs. ARFID	0.53	[−0.20, 1.26]	0.50	1.41	0.16
ANR vs. BED	0.53	[−0.37, 1.44]	0.50	1.14	0.25
ANR vs. BN	0.01	[−0.72, 0.76]	0.01	0.04	0.96
ANR vs. OSFED	0.32	[−0.39, 1.05]	0.31	0.89	0.37
ARFID vs. BED	−0.00	[−0.59, 0.59]	−0.00	−0.00	0.99
**ARFID vs. BN**	**−0.51**	**[−0.79, −0.23]**	**−0.48**	**−3.66**	**0.0003 ^†^**
ARFID vs. OSFED	−0.20	[−0.39, −0.01]	−0.19	−2.03	0.04 ^†^
BED vs. BN	−0.51	[−1.12, 0.09]	−0.48	−1.66	0.10
BED vs. OSFED	−0.20	[−0.77, 0.37]	−0.19	−0.69	0.49
BN vs. OSFED	0.31	[0.08, 0.54]	0.29	2.64	0.09 ^†^
**Psychological**					
AN-BP vs. AN-R	0.36	[−0.86, 1.59]	0.33	0.57	0.56
AN-BP vs. ARFID	−0.53	[−1.55, 0.49]	−0.49	−1.01	0.31
AN-BP vs. BED	0.42	[−0.72, 1.58]	0.40	0.73	0.47
AN-BP vs. BN	0.00	[−1.02, 1.03]	0.00	0.00	0.99
AN-BP vs. OSFED	0.11	[−0.89, 1.12]	0.10	0.22	0.82
AN-R vs. ARFID	−0.89	[−1.62, −0.15]	−0.83	−2.37	0.02 ^†^
AN-R vs. BED	0.06	[−0.84, 0.97]	0.06	0.14	0.88
AN-R vs. BN	−0.35	[−1.10, 0.38]	−0.33	−0.94	0.35
AN-R vs. OSFED	−0.24	[−0.96, 0.47]	−0.23	−0.67	0.50
**ARFID vs. BED**	**0.96**	**[0.36, 1.55]**	**0.90**	**3.18**	**0.002 ^†^**
**ARFID vs. BN**	**0.53**	**[0.25, 0.81]**	**0.50**	**3.79**	**0.0002 ^†^**
**ARFID vs. OSFED**	**0.64**	**[0.44, 0.84]**	**0.60**	**6.45**	**<0.0001 ^†^**
BED vs. BN	−0.42	[−1.03, 0.17]	−0.40	−1.38	0.17
BED vs. OSFED	−0.31	[−0.88, 0.26]	−0.29	−1.07	0.28
BN vs. OSFED	0.11	[−0.11, 0.34]	0.10	0.95	0.34
**Social Relationships**					
AN-BP vs. AN-R	0.64	[−0.58, 1.87]	0.63	1.03	0.30
AN-BP vs. ARFID	1.30	[0.28, 2.33]	1.28	2.51	0.01 ^†^
**AN-BP vs. BED**	**1.98**	**[0.82, 3.13]**	**1.94**	**3.36**	**0.0008 ^†^**
AN-BP vs. BN	1.40	[0.37, 2.43]	1.37	2.67	0.08 ^†^
AN-BP vs. OSFED	1.48	[0.47, 2.50]	1.46	2.88	0.004 ^†^
AN-R vs. ARFID	0.66	[−0.07, 1.40]	0.65	1.76	0.08
AN-R vs. BED	1.33	[0.42, 2.24]	1.31	2.87	0.004 ^†^
AN-R vs. BN	0.75	[0.01, 1.50]	0.74	1.98	0.047 ^†^
AN-R vs. OSFED	0.84	[0.12, 1.56]	0.82	2.29	0.02 ^†^
ARFID vs. BED	0.67	[0.07, 1.26]	0.65	2.22	0.03 ^†^
ARFID vs. BN	0.09	[−0.18, 0.36]	0.09	0.65	0.51
ARFID vs. OSFED	0.17	[−0.01, 0.37]	0.17	1.78	0.07
BED vs. BN	−0.57	[−1.18, 0.02]	−0.56	−1.87	0.06
BED vs. OSFED	−0.49	[−1.06, 0.08]	−0.48	−1.68	0.09
BN vs. OSFED	0.08	[−0.14, 0.31]	0.08	0.73	0.46
**Environment**					
AN-BP vs. AN-R	0.13	[−1.09, 1.361]	0.12	0.21	0.83
AN-BP vs. ARFID	0.44	[−0.58, 1.46]	0.42	0.84	0.40
AN-BP vs. BED	0.78	[−0.37, 1.93]	0.76	1.32	0.18
AN-BP vs. BN	0.06	[−0.96, 1.09]	0.06	0.12	0.90
AN-BP vs. OSFED	0.40	[−0.60, 1.41]	0.39	0.78	0.43
AN-R vs. ARFID	0.30	[−0.43, 1.04]	0.29	0.81	0.41
AN-R vs. BED	0.64	[−0.26, 1.55]	0.63	1.39	0.16
AN-R vs. BN	−0.06	[−0.81, 0.68]	−0.06	−0.17	0.86
AN-R vs. OSFED	0.27	[−0.44, 0.99]	0.26	0.74	0.46
ARFID vs. BED	0.34	[−0.25, 0.93]	0.33	1.13	0.26
ARFID vs. BN	−0.37	[−0.64, −0.09]	−0.36	−2.65	0.09 ^†^
ARFID vs. OSFED	−0.03	[−0.23, 0.16]	−0.03	−0.35	0.72
BED vs. BN	−0.71	[−1.32, −0.10]	−0.69	−2.31	0.02 ^†^
BED vs. OSFED	−0.37	[−0.95, 0.19]	−0.36	−1.28	0.20
BN vs. OSFED	0.33	[0.10, 0.56]	0.32	2.86	0.004 ^†^

Note. AN-R = anorexia nervosa-restricting subtype; AN-BP = anorexia nervosa-binge/purge subtype; BN = bulimia nervosa; BED = binge eating disorder; ARFID = avoidant/restrictive food intake disorder; OSFED = other specified feeding or eating disorder; BMI = body mass index. One model was run for each outcome, with eating disorder (ED) diagnosis as a six-level dummy-coded variable, producing 15 total pairwise contrasts for each model. All models were adjusted for age, sex, level of care, BMI, and the interaction between BMI and ED diagnosis. Bolded comparisons remained significant after Holm multiple comparison correction. ^†^ Comparison statistically significant before Holm multiple comparison correction. Cohen’s *d* effect sizes represent differences in marginal means, when accounting for covariates, over residual variance (which was equivalent to standard unadjusted pooled variance), and therefore is not directly comparable to unadjusted Cohen’s *d*’s.

**Table 3:** Unadjusted Pairwise Comparisons of Quality of Life Between Eating Disorder Diagnoses.

WHOQOL Scale	Cohen's *d*	*d* 95% CI	*b*-Value	*t*-Value	*p*-Value
**Overall**					
AN-BP vs. AN-R	−0.06	[−0.27, 0.15]	−0.06	−0.56	0.57
AN-BP vs. ARFID	0.01	[−0.22, 0.24]	0.01	0.05	0.96
AN-BP vs. BED	0.30	[−0.06, 0.67]	0.30	1.62	0.11
AN-BP vs. BN	−0.18	[−0.44, 0.07]	−0.18	−1.37	0.17
AN-BP vs. OSFED	−0.02	[−0.22, 0.16]	−0.02	−0.29	0.76
AN-R vs. ARFID	0.06	[−0.12, 0.26]	0.06	0.68	0.49
AN-R vs. BED	0.36	[0.01, 0.71]	0.36	2.07	0.04 ^†^
AN-R vs. BN	−0.12	[−0.35, 0.10]	−0.12	−1.03	0.30
AN-R vs. OSFED	0.03	[−0.11, 0.18]	0.03	0.43	0.66
ARFID vs. BED	0.30	[−0.06, 0.66]	0.30	1.62	0.10
ARFID vs. BN	−0.19	[−0.43, 0.05]	−0.19	−1.50	0.13
ARFID vs. OSFED	−0.03	[−0.21, 0.13]	−0.03	−0.40	0.69
BED vs. BN	−0.49	[−0.87, −0.11]	−0.49	−2.53	0.01 ^†^
BED vs. OSFED	−0.33	[−0.67, 0.0003]	−0.33	−1.96	0.0501
BN vs. OSFED	0.15	[−0.05, 0.36]	0.15	1.43	0.15
**Physical Health**					
AN-BP vs. AN-R	−0.15	[−0.36, 0.06]	−0.15	−1.39	0.16
AN-BP vs. ARFID	0.14	[−0.09, 0.38]	0.14	1.20	0.23
AN-BP vs. BED	0.27	[−0.09, 0.64]	0.27	1.45	0.15
AN-BP vs. BN	−0.17	[−0.44, 0.08]	−0.17	−1.33	0.18
AN-BP vs. OSFED	0.08	[−0.11, 0.27]	0.08	0.82	0.41
**AN-R vs. ARFID**	**0.29**	**[0.09, 0.49]**	**0.29**	**2.95**	**0.003 ^†^**
AN-R vs. BED	0.42	[0.07, 0.77]	0.42	2.40	0.02 ^†^
AN-R vs. BN	−0.02	[−0.25, 0.20]	−0.02	−0.22	0.82
**AN-R vs. OSFED**	**0.23**	**[0.08, 0.38]**	**0.23**	**3.12**	**0.002 ^†^**
ARFID vs. BED	0.12	[−0.23, 0.49]	0.12	0.70	0.48
ARFID vs. BN	−0.32	[−0.57, −0.07]	−0.32	−2.55	0.01 ^†^
ARFID vs. OSFED	−0.06	[−0.23, 0.11]	−0.06	−0.71	0.48
BED vs. BN	−0.45	[−0.83, −0.07]	−0.45	−2.34	0.02 ^†^
BED vs. OSFED	−0.19	[−0.53, 0.14]	−0.19	−1.12	0.26
BN vs. OSFED	0.26	[0.05, 0.47]	0.25	2.43	0.02^†^
**Psychological**					
AN-BP vs. AN-R	−0.28	[−0.50, −0.07]	−0.28	−2.62	0.09
**AN-BP vs. ARFID**	**−0.73**	**[−0.96, −0.49]**	**−0.71**	**−6.09**	**<.0001 ^†^**
AN-BP vs. BED	−0.14	[−0.51, 0.22]	−0.14	−0.77	0.44
AN-BP vs. BN	−0.15	[−0.42, 0.10]	−0.15	−1.17	0.24
AN-BP vs. OSFED	−0.00	[−0.19, 0.19]	−0.00	−0.01	0.98
**AN-R vs. ARFID**	**−0.44**	**[−0.64, −0.24]**	**−0.43**	**−4.37**	**<.0001 ^†^**
AN-R vs. BED	0.14	[−0.20, 0.49]	0.13	0.79	0.43
AN-R vs. BN	0.13	[−0.09, 0.36]	0.12	1.11	0.26
**AN-R vs. OSFED**	**0.28**	**[0.13, 0.43]**	**0.27**	**3.82**	**0.0001 ^†^**
**ARFID vs. BED**	**0.58**	**[0.22, 0.94]**	**0.56**	**3.16**	**0.0016 ^†^**
**ARFID vs. BN**	**0.57**	**[0.32, 0.82]**	**0.55**	**4.52**	**<.0001 ^†^**
**ARFID vs. OSFED**	**0.72**	**[0.55, 0.90]**	**0.70**	**8.18**	**<.0001 ^†^**
BED vs. BN	−0.01	[−0.39, 0.36]	−0.01	−0.05	0.95
BED vs. OSFED	0.14	[−0.19, 0.48]	0.14	0.84	0.40
BN vs. OSFED	0.15	[−0.05, 0.36]	0.15	1.45	0.15
**Social Relationships**					
AN-BP vs. AN-R	−0.11	[−0.33, 0.09]	−0.11	−1.06	0.29
AN-BP vs. ARFID	−0.09	[−0.33, 0.13]	−0.09	−0.80	0.42
AN-BP vs. BED	0.46	[0.09, 0.83]	0.46	2.46	0.01^†^
AN-BP vs. BN	−0.12	[−0.39, 0.13]	−0.12	−0.96	0.34
AN-BP vs. OSFED	0.02	[−0.16, 0.22]	0.02	0.26	0.79
AN-R vs. ARFID	0.02	[−0.17, 0.21]	0.02	0.20	0.84
**AN-R vs. BED**	**0.58**	**[0.23, 0.93]**	**0.58**	**3.27**	**0.001 ^†^**
AN-R vs. BN	−0.01	[−0.24, 0.21]	−0.01	−0.09	0.92
AN-R vs. OSFED	0.14	[−0.00, 0.29]	0.14	1.91	0.06
**ARFID vs. BED**	**0.56**	**[0.20, 0.92]**	**0.56**	**3.05**	**0.002 ^†^**
ARFID vs. BN	−0.03	[−0.28, 0.21]	−0.03	−0.25	0.80
ARFID vs. OSFED	0.12	[−0.05, 0.29]	0.12	1.37	0.17
**BED vs. BN**	**−0.59**	**[−0.97, −0.21]**	**−0.59**	**−3.07**	**0.002 ^†^**
BED vs. OSFED	−0.44	[−0.77, −0.10]	−0.43	−2.56	0.01 ^†^
BN vs. OSFED	0.15	[−0.05, 0.36]	0.15	1.44	0.15
**Environment**					
AN-BP vs. AN-R	−0.21	[−0.42, 0.01]	−0.20	−1.91	0.06
AN-BP vs. ARFID	0.07	[−0.15, 0.31]	0.07	0.63	0.53
AN-BP vs. BED	0.12	[−0.24, 0.49]	0.12	0.64	0.52
AN-BP vs. BN	−0.18	[−0.45, 0.07]	−0.18	−1.41	0.16
AN-BP vs. OSFED	0.14	[−0.05, 0.33]	0.14	1.43	0.15
AN-R vs. ARFID	0.28	[0.08, 0.48]	0.28	2.83	0.005 ^†^
AN-R vs. BED	0.33	[−0.01, 0.68]	0.33	1.87	0.06
AN-R vs. BN	0.02	[−0.20, 0.25]	0.02	0.17	0.86
**AN-R vs. OSFED**	**0.35**	**[0.20, 0.50]**	**0.34**	**4.70**	**<.0001 ^†^**
ARFID vs. BED	0.04	[−0.31, 0.40]	0.04	0.25	0.80
ARFID vs. BN	−0.26	[−0.51, −0.01]	−0.26	−2.09	0.04 ^†^
ARFID vs. OSFED	0.06	[−0.10, 0.24]	0.06	0.74	0.45
BED vs. BN	−0.31	[−0.69, 0.06]	−0.30	−1.61	0.11
BED vs. OSFED	0.01	[−0.31, 0.35]	0.01	0.11	0.91
**BN vs. OSFED**	**0.33**	**[0.12, 0.54]**	**0.32**	**3.09**	**0.002 ^†^**

Note. AN-R = anorexia nervosa-restricting subtype; AN-BP = anorexia nervosa-binge/purge subtype; BN = bulimia nervosa; BED = binge eating disorder; ARFID = avoidant/restrictive food intake disorder; OSFED = other specified feeding or eating disorder; BMI = body mass index. One model was run for each outcome, with eating disorder (ED) diagnosis as a 6-level dummy-coded variable, producing 15 total pairwise contrasts for each model. All models unadjusted. Bolded comparison remained significant after Holm multiple comparison correction. ^†^ Comparison statistically significant before Holm multiple comparison correction.

**Table 4:** Rank-Ordered WHOQOL-BREF Scores by Diagnosis.

WHOQOL-BREF Scale	Mean	SD
**Overall**		
BN	5.62	1.71
AN-R	5.40	1.71
OSFED	5.35	1.82
AN-BP	5.29	1.79
ARFID	5.28	1.71
BED	4.75	1.68
**Physical Health**		
BN	22.8	5.46
AN-R	22.7	5.39
AN-BP	21.8	5.64
OSFED	21.4	5.34
ARFID	21.1	5.31
BED	20.4	5.79
**Psychological**		
ARFID	17.0	3.80
AN-R	15.1	4.46
BN	14.5	3.78
BED	14.5	4.51
OSFED	13.9	4.18
AN-BP	13.8	5.14
**Social Relationships**		
BN	9.76	2.49
AN-R	9.74	2.57
ARFID	9.68	2.45
AN-BP	9.43	2.73
OSFED	9.36	2.63
BED	8.22	2.58
**Environment**		
AN-R	30.8	5.86
BN	30.6	5.50
AN-BP	29.5	5.86
ARFID	29.1	5.30
BED	28.8	7.14
OSFED	28.7	6.10

Note. AN-R = anorexia nervosa-restricting subtype; AN-BP = anorexia nervosa-binge/purge subtype; BN = bulimia nervosa; BED = binge eating disorder; ARFID = avoidant/restrictive food intake disorder; OSFED = other specified feeding or eating disorder.

## Data Availability

The data presented in this article are not readily available due to privacy reasons. Requests should be sent to the corresponding author.

## Supplementary Materials

The following supporting information can be downloaded at: https://www.mdpi.com/article/10.3390/bs16071174/s1, Figure S1: Adjusted Pairwise Comparisons of Quality of Life Between ED Diagnoses (Cohen’s d with 95% Cl); Figure S2: Unadjusted Pairwise Comparisons of Quality of Life Between ED Diagnoses (Cohen’s d with 95% Cl); Figure S3: Rank-Ordered WHOQOL-BREF Scores by Diagnosis.
