# Supplementary figures and images for: Quality of Life in Adults with Eating Disorders
Source: Behav Sci (Basel). 2026 Jul 11;16(7):1174. doi: 10.3390/bs16071174 (PMC13403506; doi:10.3390/bs16071174)

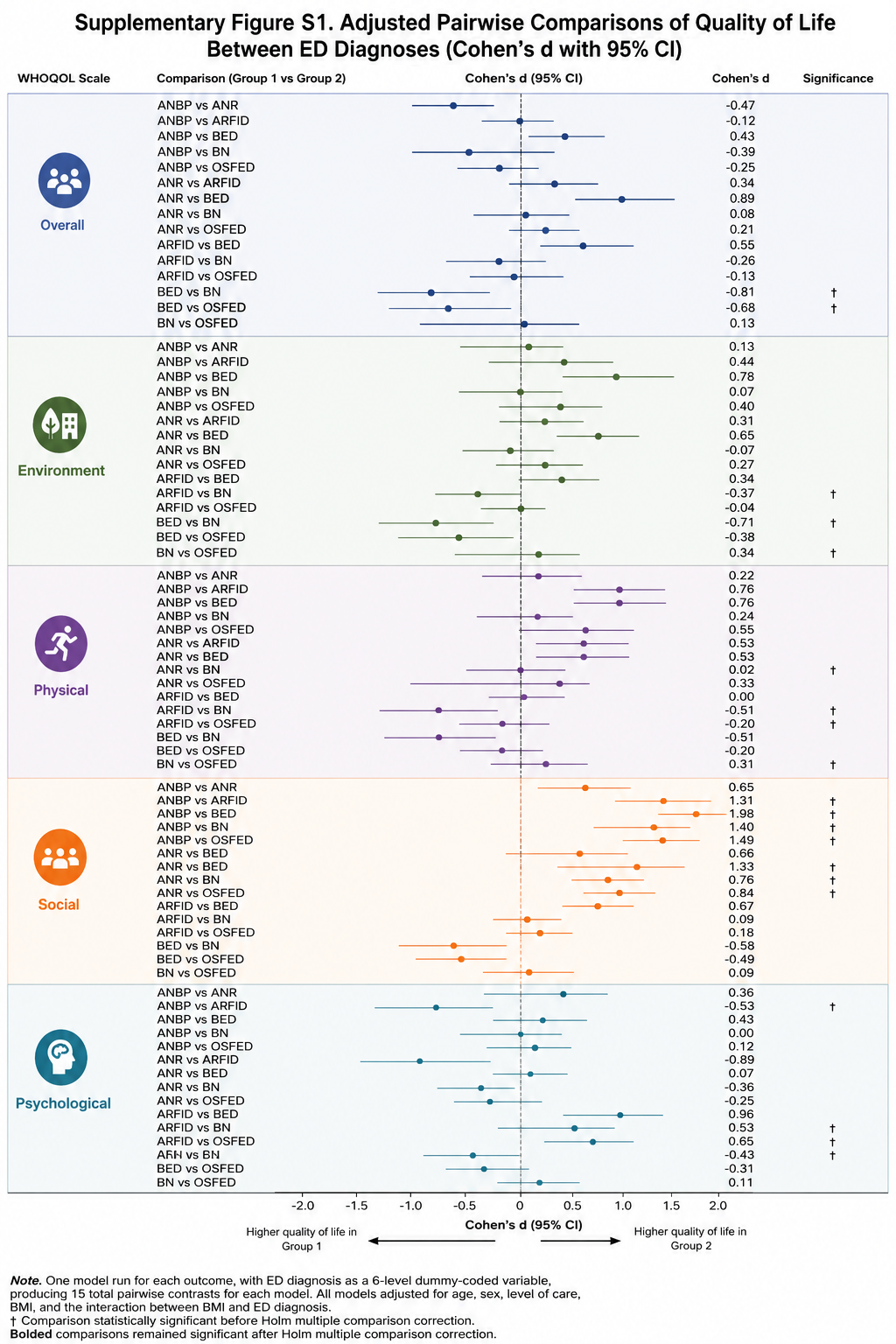

Supplement: Supplementary file 1 [file behavsci-16-01174-s001.zip › SupplementalFigureS1_WHOQOL.png]

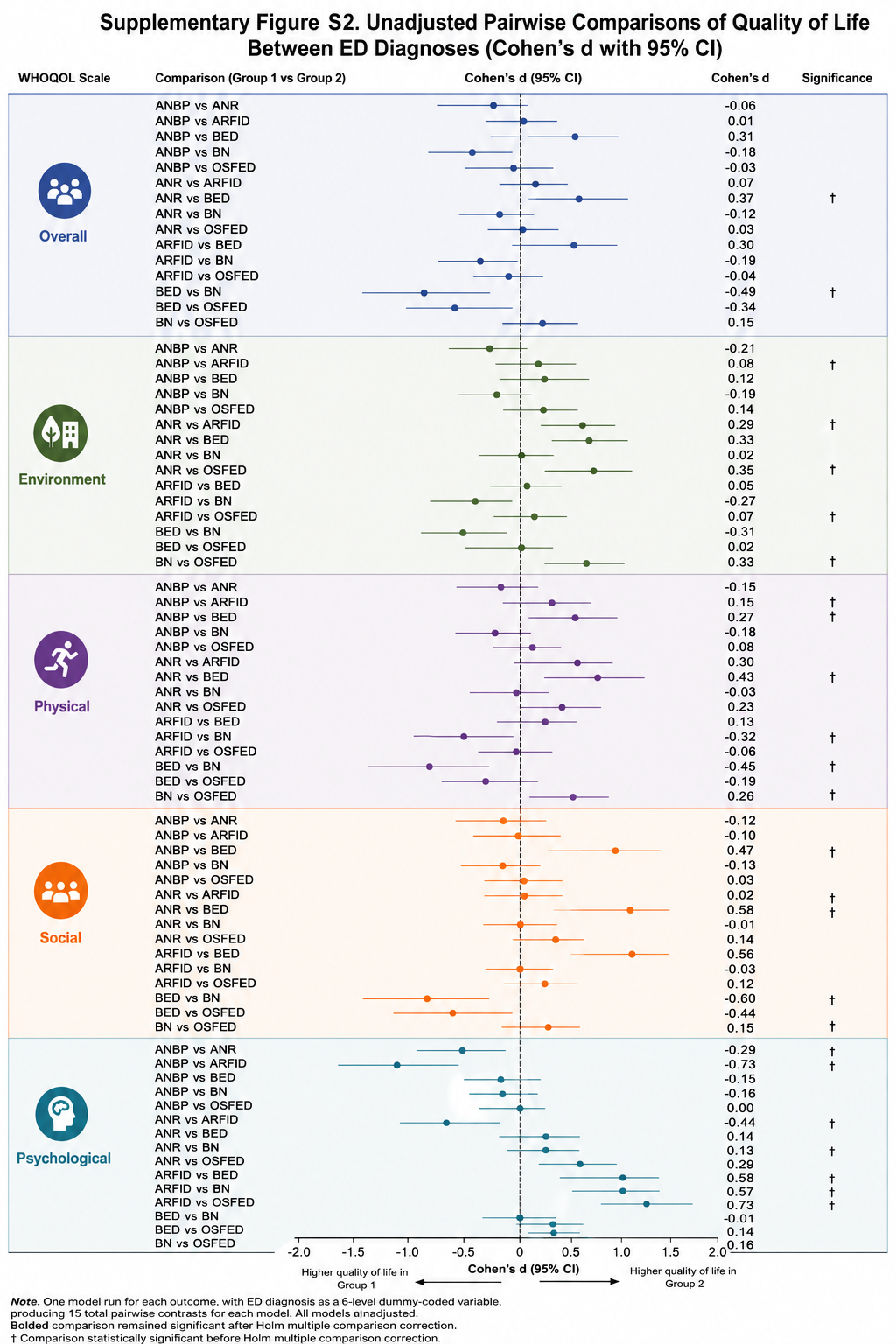

Supplement: Supplementary file 1 [file behavsci-16-01174-s001.zip › SupplementalFigureS2_WHOQOL.png]

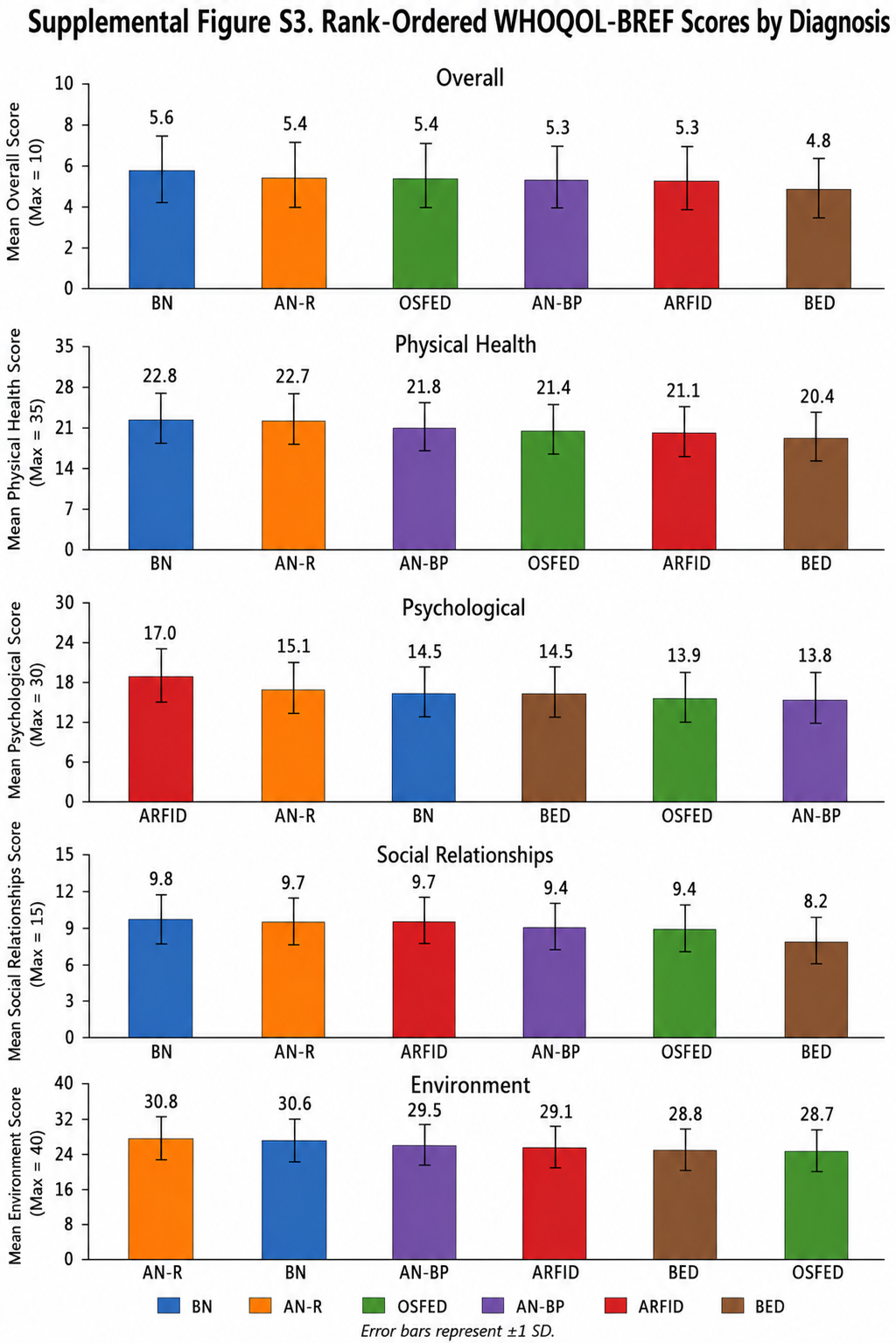

Supplement: Supplementary file 1 [file behavsci-16-01174-s001.zip › SupplementalFigureS3_WHOQOL.png]
